# The First-Night Effect in Elite Sports: An Initial Glance on Polysomnography in Home-Based Settings

**DOI:** 10.3389/fpsyg.2021.641451

**Published:** 2021-03-25

**Authors:** Annika Hof zum Berge, Michael Kellmann, Sarah Jakowski

**Affiliations:** ^1^Faculty of Sport Science, Unit of Sport Psychology, Ruhr University Bochum, Bochum, Germany; ^2^School of Human Movement and Nutrition Sciences, The University of Queensland, St. Lucia, QLD, Australia

**Keywords:** sleep, portable PSG, insomnia, athlete, monitoring, somnology

## Abstract

Self-applied portable polysomnography is considered a promising tool to assess sleep architecture in field studies. However, no findings have been published regarding the appearance of a first-night effect within a sport-specific setting. Its absence, however, would allow for a single night sleep monitoring and hence minimize the burden on athletes while still obtaining the most important variables. For this reason, the aim of the study was to assess whether the effect appears in home-based sleep monitoring of elite athletes.

The study sample included eight male and 12 female German elite athletes from five different sports. Participants slept with a portable polysomnography for two nights, which they self-applied at night before going to bed. Time in bed and wake-up time in the morning were freely chosen by each individual athlete without any restrictions regarding time or sleeping environment. Participants were asked to keep the same location and time frame during the two days of monitoring and stick to their usual sleeping schedules. Sleep stages were manually scored using 30-s epochs. Sleep parameters and stages were later compared with the help of linear mixed models to investigate the factor time.

Significant differences between the two nights were found for percentage of Non-REM sleep [T(19) = −2,10, *p* < 0.05, *d* = −0.47, 95%-CI (−7.23, −0.01)] with small effect size, Total Wake Time [T(19) = 2.30, *p* = 0.03, *d* = 0.51, 95%-CI (1.66, 35.17)], Sleep Efficiency [T(19) = −2.48, *p* = 0.02, *d* = −0.55, 95%-CI (−7.43, −0.63)], and Wake percentage [T(19) = 2.47, *p* = 0.02, *d* = 0.55, 95%-CI (0.61, 7.43)] with moderate effect sizes, and N3 Sleep Onset Latency [T(19) = 3.37, *p* < 0.01, *d* = 0.75, 95%-CI (7.15, 30.54)] with large effect size. Confidence Intervals for all other indices range from negative to positive values and hence specify, that parameters were not systematically negatively affected in the first night.

Findings suggest that some individuals are more affected by the first-night effect than others. Yet, in order to keep the measurement uncertainties to a minimum, a more conservative approach with at least two monitoring nights should be used whenever possible, if no other supporting information on the athletes says otherwise.

## Introduction

Sleep is not only one of the most popular methods for recovery in elite athletes (Venter, 2014), but also considered vital to human health and well-being, and critical to physiological and cognitive functioning (Horne, 1988). Within a night of sleep, the different sleep stages are linked to different physiological and psychological effects on the human body. While Slow Wave Sleep (SWS), also called deep sleep, is believed to have a mostly physiological effect on recovering body and muscles, Rapid-Eye-Movement (REM) sleep stage is believed to have a regulating effect on memory and emotion processing. Within one night, a healthy sleeper passes through between five and seven sleep cycles of around 90 min each. In the first half of the night, a sleep cycle mostly consists of light sleep (N1 and N2) and deep sleep (N3), so called Non-REM sleep cycles. In the second half of the night, deep sleep is reduced and REM sleep is primarily occurring (Dotto, 1996; Carskadon and Dement, 2011). An absence of the recommended amount of sleep is associated with several adverse psychological and physiological outcomes, including impaired cognitive and physical performance (Dinges et al., 1997; Van Dongen et al., 2003; Bolin, 2019). Specifically, inadequate sleep in healthy populations is linked with impairments to accuracy, reaction time, and decision making (Smith et al., 2019; Vincent et al., 2020). While the demands in elite sports are ever increasing, the role of adequate recovery becomes a fundamental factor (Caia et al., 2018; Lalor et al., 2020). Given the abundant complex neurophysiological relationships that can influence learning processes and long-term memory, it prospectively plays a vital role in the development of technical, tactical, and physiological aspects of sport performance (Fullagar et al., 2020).

However, sleep is a rather challenging matter in elite sports (Caia et al., 2018; Kölling et al., 2019). Multiple internal and external circumstances negatively affect an athlete's sleep quality and quantity and can be determined as salient risk factors for sleep inadequacy in athletes (Kölling et al., 2016b; Lastella et al., 2020; Walsh et al., 2020). For one thing, international air travel is required for athletes to get to different competition grounds or training camps. This way they face different time zones and different sleeping conditions, often both at the same time. A study by Fowler et al. (2015) indicates that sleep disruption resulting from conditions during travel may increase perceptual fatigue and suppress lower-body power. On the other hand, influences on sleep duration and quality may be late-night start-up, bright stadium lighting, and result-dependent mood; all factors which might influence an athlete's circadian rhythm (Fullagar et al., 2016). Nevertheless, sleep deprivation cannot only be found during competition. Early morning and late-night training sessions may affect the sleep duration (Sargent et al., 2014), especially for those athletes still going to school or working in-between sessions. However, available research only scratches the surface on how sleep influences athlete health (Walsh et al., 2020). As sleep quality may vary highly, even within a single team, there is an urgent need for highly individual sleep intervention programs (Hof zum Berge et al., 2021). Consequently, a one-size-fits-all approach for elite athletes is unlikely ideal for health and performance (Walsh et al., 2020). Hence, recent research highly stresses the importance of the implementation of an individual sleep monitoring into an athlete's routine to further investigate sleep in the elite athlete population (Kellmann et al., 2018; O'Donnell et al., 2018; Halson, 2019; Fullagar et al., 2020; Walsh et al., 2020). Yet, there are several approaches to monitor sleep and the assessment tool must always be tailored toward the specific purpose of measurement (Martin and Hakim, 2011; Hof zum Berge et al., 2020b).

In sport-scientific studies, sleep data is mostly obtained by using subjective questionnaires or wrist actigraphy (Sargent et al., 2018; Simim et al., 2020). Benefits of the wrist actigraphy are its non-invasiveness, portability, easy accessibility for athletes, and usage in ordinary sleeping environments. However, sleep latency, sleep efficiency, and wake episodes after sleep onset should be interpreted cautiously, as the (lack of) movement that occurs during sleep can be mistaken for either wake or sleep. This, in turn may lead to over- or underestimation of sleep indices (O'Donnell et al., 2018). Further, wrist actigraphy neglects the assessment of sleep stages. Thus, although wrist-actigraphy may be a highly functional tool for long-term sleep monitoring of general sleep patterns and behaviors, it may not be suitable to examine, or monitor interventions on sleep quality issues, such as trouble falling asleep or not feeling rested after a non-restricted night of sleep (Halson, 2019; Hof zum Berge et al., 2020b). A different approach is to use mobile applications to monitor sleep. First applications on the market promise to monitor sleep stages via acceleration and microphone; however, they do not result sufficiently reliable in sleep–wake detection. Rather, they exhibit significant differences in sleep stages identification, while simultaneously underestimating wake and sleep efficiency and overestimating deep sleep and total sleep time when compared with in-lab polysomnography (Fino et al., 2020; Hof zum Berge et al., 2020a).

This leads to polysomnography (PSG) still being the gold-standard tool for sleep assessment (Rechtschaffen and Kales, 1968). It is commonly used in a stationary sleep-lab with supervision during the night, allowing for high quality data (Kölling et al., 2016a; Hof zum Berge et al., 2020b). Yet, there are some confounders when assessing sleep in a lab. The so-called first-night effect for instance, implies that sleep architecture is deteriorated during sleep assessment. Accordingly, the first-night effect is mainly characterized by lower sleep efficiency, increased wakefulness, reduction in the amount of REM sleep, and longer sleep and REM sleep latencies (Agnew et al., 1966). The cause of the first-night effect is most likely multifactorial, including discomfort caused by electrodes, limitation of movements by gauges and cables as well as potential psychological consequences of being under examination and experiencing a new sleeping environment. Up to this point, the first-night effect remains a decisive topic in sleep studies since it might bias any PSG performed for clinical or research purposes (Le Bon et al., 2001). Nonetheless, sleep quality improves significantly during the second night, when testing young and healthy participants (Agnew et al., 1966; Lorenzo and Barbanoj, 2002; Tamaki et al., 2014). For this reason, the integration of a baseline session before the experimental sleep sessions has been advocated in order to allow for participants' adaptation to the new sleep environment (Tamaki et al., 2005). Nowadays, advanced technology allows for portable PSG without the necessity to sleep in an unfamiliar sleep setting. It is a promising tool to assess not only sleep quantity, but also sleep architecture in elite athletes without having them visit a sleep-lab (Hof zum Berge et al., 2020b). While clinical diagnosis must still be overlooked in a stationary sleep-lab, portable PSG occurs to be an efficient and reliable gateway option that can fill the void and enable new research on the sleep stage distribution in elite athletes to later develop hands-on intervention programs (Hof zum Berge et al., 2020a).

Yet, while no first-night effect appears to be found in actigraphy (Van Hilten et al., 1993; Jean-Louis et al., 1996; Driller and Dunican, 2020), no findings have been published up to this point regarding the appearance of the first-night effect in portable PSG in sport settings (Hof zum Berge et al., 2020a,b). However, as athletes are exposed to a variety of different stressors, the main goal of monitoring is to keep investment for the athletes to a minimum to gather as much important information as necessary in the shortest time and with the least stress possible. Consequently, the absence of the first-night effect in portable PSG would allow for a single night sleep monitoring to assess sleep architecture in an athlete. Though, other confounders as discomfort, movement range, and psychological stressors may still appear. Several studies have examined the appearance of the first-night effect in home-based settings with contradicting results of whether a first-night effect may be found (Le Bon et al., 2001; Abumuamar et al., 2018; Miettinen et al., 2018). Hence, there is no general consent whether the first-night effect in home-based settings exists, but rather different results for different target groups and research set-ups. For this reason, the aim of the study is to assess whether the first-night effect appears in in-home sleep monitoring of elite athletes.

## Materials and Methods

### Participants

The study sample included eight male and 12 female German elite athletes (age = 21.70 ± 4.53 years*;* height = 172.35 ± 7.41 cm*;* weight = 68.53 ± 14.02 kg) from five different sports (equestrian vaulting, *n* = 5; paddle tennis, *n* = 3; karate, *n* = 2; athletics, *n* = 5; soccer, *n* = 5), all being members of either the junior (karate, athletics) or the senior (paddle tennis, equestrian vaulting) national team of their sporting federation or playing in national first division (soccer). Participants were drawn from a large-scaled database assessing sleep patterns in different sporting disciplines. Athletes were offered to undergo a voluntary two-night sleep assessment via portable PSG. Those athletes who agreed to take part in this monitoring were included in the sub-dataset presented in this manuscript. Ethical clearance for the study was obtained by the local ethics committee ahead of assessment in accordance with the declaration of Helsinki. All participants were briefed about the aims of the study, selected regarding possible exclusion criteria (i.e., neurological disorders or use of sleep-influencing medication) and signed an informed written consent. For minors, legal guardians gave additional consent to the participation in the study. At study completion, participants received a written individual interpretation of their sleep data constructed by a sleep medicine expert.

### Procedure

Participants slept with portable PSG for consecutive of two nights (SOMNOwatch plus EEG, SOMNOmedics GmbH, Randersacker, Germany). With a 97.79% sensitivity and accuracy of 97.06% of the epochs being correctly identified, the SOMNOwatch plus EEG serves as a reliable tool for long-term recorded total sleep time evaluation (Voinescu et al., 2014). Further, a first study by Hof zum Berge et al. (2020b) advocates the SOMNOwatch plus EEG device to be a suitable and self-applicable device whenever information cannot be sufficiently achieved by actigraphy. Participants were informed about the handling of the device in a standardized personal briefing and received additional picture- and video-based instructions as a further guideline. For application of the SOMNOwatch plus EEG device, ten self-adhesive silver chloride electrodes (Fiab, Florence, Italy) were self-applied (F3, F4, A1, A2, two EOGs, two EMGs, aFz, FCZ) and digitized with a sampling frequency of 256 Hz. Sleep stages were manually scored by one individual scorer using 30-s epochs according to the American Academy of Sleep Medicine (AASM) guidelines (Berry et al., 2012) with adaptations to the single-EEG-device based on the criteria suggested by Lucey et al. (2016). Time in bed and wake-up time in the morning were freely chosen by each individual athlete without any restrictions regarding time or sleeping environment, but they were asked to keep the same location and time frame during the two days of monitoring and stick to their usual sleeping schedules.

### Statistical Analysis

Statistical analyses were performed using SPSS V.25 (IBM Corporation; Chicago, IL, USA). Level of two-tailed significance was set to *p* < 0.05. Descriptive statistics are continuously presented as means ± standard deviation. Sleep parameters Sleep Onset Latency (SOL; duration of falling asleep, in minutes), Wake After Sleep Onset (WASO; amount of time spent awake after falling asleep, in minutes), Total Wake Time (TWT; amount of actual wake time, in minutes), Total Sleep Time (TST; amount of actual sleep time, in minutes), Sleep Efficiency (SE; TST divided by Time in Bed and multiplied by 100), and the sleep stages (N1, N2, N3, REM) of the two nights (fixed factor *time*) were compared using linear mixed model analysis, as recommended by Boisgontier and Cheval (2016). Within the analysis, sleep parameters and stages each pitch as a function of time (sleep indices ~ time). Further, Cohen's effect sizes (*d*) were calculated and interpreted using thresholds of 0.2, 0.5, 0.8 for small, moderate, and large, respectively (Cohen, 1988).

## Results

Overall, athletes spend an average of 7 h and 47 min (±1:20) in bed (TIB), of which they slept 6 h and 45 min (±1:11; TST). It took them 15 (±26) min to fall asleep (SOL), and 1 h and 38 min (±0:46) to first hit deep sleep (SOL N3). An overview of all sleep parameters for the two nights is presented in Table 1.

**Table 1:** Overview of sleep parameters (in minutes) averaged over both nights (*n* = 40).

	**Min**	**Max**	***M***	***SD***
TIB	257.18	597.12	466.89	80.14
TST	226.40	549.00	405.16	71.30
TWT	15.09	175.70	61.59	33.66
SOL	0.00	147.32	15.76	26.66
REM SOL	6.00	213.00	97.38	46.35
N3 SOL	6.83	183.82	37.15	33.98

*TIB, Time in Bed; TST, Total Sleep Time; TWT, Total Wake Time; REM, Rapid Eye Movement; SOL, Sleep Onset Latency; n, number of nights; M, Mean; Min, Minimum; Max, Maximum; SD, standard deviation*.

Participants averagely slept 86.99 percent of the time in bed (SE). Within the night, 68.97 percent consisted of Non-REM and 18.02 percent consisted of REM sleep. Non-REM sleep was further divided into light sleep, with 42.51 percent of the night, and deep sleep, with 26.45 percent of the night. An overview of the sleep stage distribution can be drawn from Table 2.

**Table 2:** Overview of sleep stages (in percent) averaged over both nights (*n* = 40).

	**Min**	**Max**	***M***	***SD***
Wake percentage	3.55	33.23	12.98	6.26
REM percentage	5.29	28.68	18.02	5.18
Non-REM percentage	45.39	87.78	68.97	7.69
N1 percentage	0.70	16.00	5.73	3.41
N2 percentage	17.59	49.52	36.78	7.67
N3 percentage	16.72	43.70	26.45	6.13
Sleep Efficiency	66.77	96.09	86.99	6.24

*REM, Rapid Eye Movement; N1, sleep stage 1; N2, sleep stage 2; N3, sleep stage 3; n, number of nights; M, Mean; Min, Minimum; Max, Maximum; SD, standard deviation*.

Estimates show that it took participants 12 (±35) min longer to fall asleep in night 1 (SOL), while they slept 27 (±84) min less (TST). Consequently, SE increased by 4.03 (±7.26) percent in the second night. All other estimates for the sleep indices can be drawn from Tables 3 and 4.

**Table 3:** Overview of linear mixed models analysis for sleep indices (in minutes) ~ time.

	**Estimates**	**SD**	***df***	***T***	***p***	**Lower-95 CI**	**Upper-95 CI**
TIB	−9.51	19.92	19	−0.477	0.639	−51.21	32.19
TST	−27.93	18.94	19	−1.475	0.157	−67.56	11.71
TWT	18.42	8.00	19	2.301	0.033^*^	1.66	35.17
SOL	12.03	7.85	19	1.533	0.142	−4.40	28.46
REM SOL	0.04	11.41	19	0.003	0.997	−23.83	23.91
N3 SOL	18.85	5.59	19	3.374	0.003^**^	7.15	30.54

*TIB, Time in Bed; TST, Total Sleep Time; TWT, Total Wake Time; REM, Rapid Eye Movement; SOL, Sleep Onset Latency; N3, sleep stage 3; SD, standard deviation; df, degrees of freedom; T, t-Value; p, level of significance*;

* *p < 0.05*;

** *p < 0.01; CI, Confidence Interval*.

**Table 4:** Overview of linear mixed models analysis for sleep indices (in percent) ~ time.

	**Estimates**	**SD**	***df***	***T***	***p***	**Lower-95 CI**	**Upper-95 CI**
Wake	4.02	1.63	19	2.472	0.023^*^	0.61	7.43
REM	−0.41	1.39	19	−0.294	0.772	−3.32	2.50
Non-REM	−3.62	1.72	19	−2.100	0.049^*^	−7.23	−0.01
N1	−0.47	1.01	19	−0.466	0.647	−2.58	1.64
N2	−1.47	2.00	19	−0.731	0.473	−5.66	2.73
N3	−1.69	1.51	19	−1.116	0.278	−4.85	1.48
Sleep Efficiency	−4.03	1.62	19	−2.481	0.023*	−7.43	−0.63

*TIB, Time in Bed; TST, Total Sleep Time; TWT, Total Wake Time; REM, Rapid Eye Movement; SOL, Sleep Onset Latency; N3, sleep stage 3; SD, standard deviation; df, degrees of freedom; T, t-Value; p, level of significance*;

* *p < 0.05; CI, Confidence Interval*.

**Figure 1:**
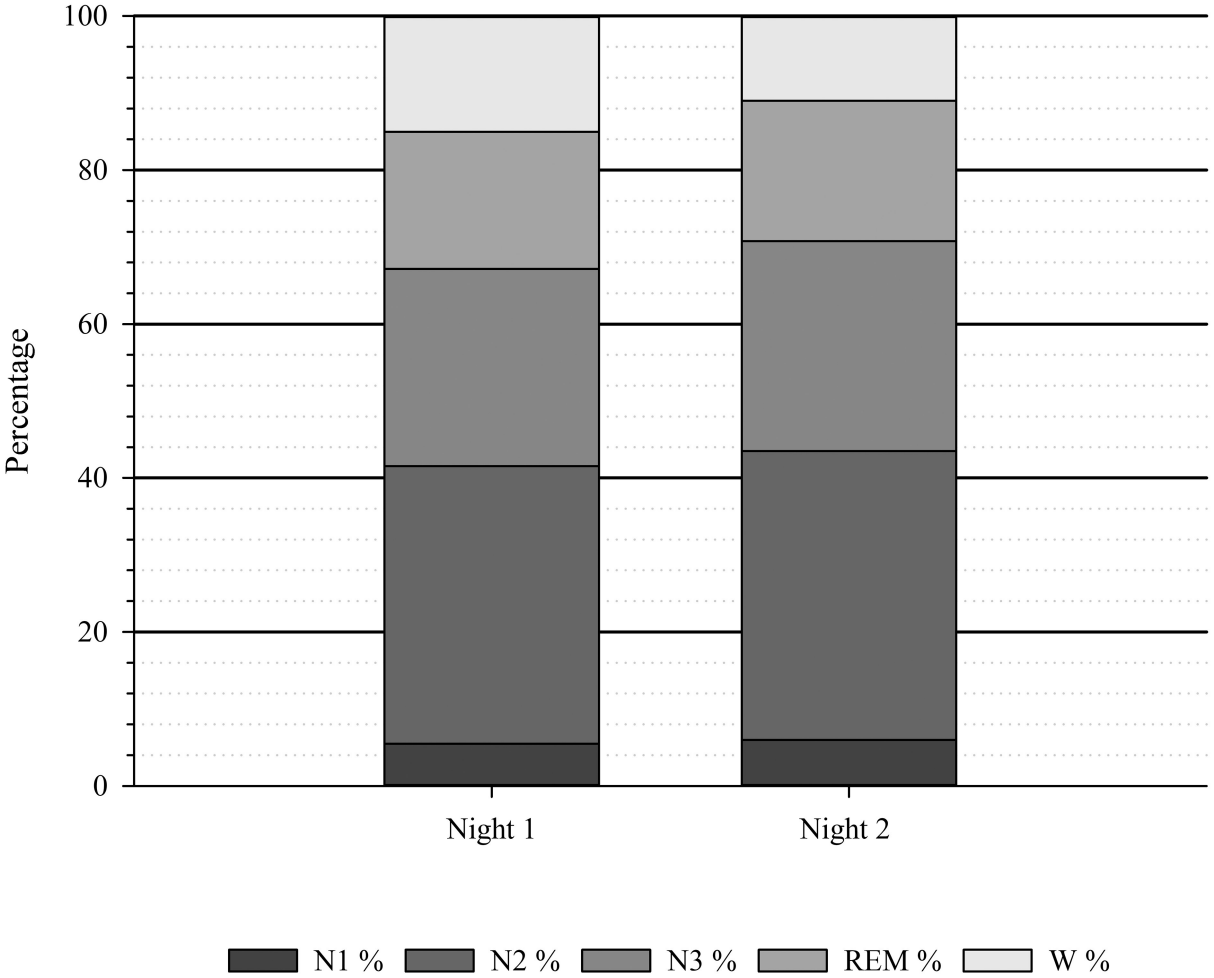
Average sleep stage distribution (*N* = 20). *N*, number of participants; N1, sleep stage 1; N2, sleep stage 2; REM, Rapid Eye Movement; W, Wake; %, percentage.

An overview of the sleep stage distribution for night one and night two is visualized in Figure 1.

Significant differences between the two nights were found for percentage of Non-REM sleep [T(19) = −2,10, *p* < 0.05, *d* = −0.47, 95%-CI (−7.23, −0.01)] with small effect size, TWT [T(19) = 2.30, *p* = 0.03, *d* = 0.51, 95%-CI (1.66, 35.17)], SE [T(19) = −2.48, *p* = 0.02, *d* = −0.55, 95%-CI (−7.43, −0.63)] and Wake percentage [T(19) = 2.47, *p* = 0.02, *d* = 0.55, 95%-CI (0.61, 7.43)] with moderate effect sizes, and N3 SOL [T(19) = 3.37, *p* < 0.01, *d* = 0.75, 95%-CI (7.15, 30.54)] with large effect size. Confidence Intervals for all other indices range from negative to positive values and hence specify, that parameters were not systematically negatively affected in the first night.

On an individual level, the shortest time spend in bed was 5:46 h (SE = 88.44%) on the first night, and 4:17 h (SE = 88.03%) on the second night, both measured for the same participant. Lowest sleep efficiency measured was 66.77% on the first night which increased to 85.27% on night two for this participant. On night two, the lowest sleep efficiency was measured at 81.33%. Six participants decreased their values of sleep efficiency in the second night with values ranging between −0.41% and −5.92%. All other participants increased their values within the second night, with changes ranging from 1.19 to 18.51%. There was no participant who did not sleep at all and no individual did not reach all sleep stages during the nights of assessment.

## Discussion

The aim of the study was to determine if a first-night effect can be found when assessing sleep in an athlete's population within home-set sleep monitoring with portable PSG. Findings suggest that average sleep quantity was reduced during the first night of sleep assessment, mostly because sleep onset was found to be delayed. Further, the findings of a significant higher occurrence of Non-REM sleep, waking periods, and the delayed onset of SWS, align with the overall characteristics of the first-night effect as it was first described by Agnew et al. (1966) and the findings for general population in home-based settings (Le Bon et al., 2001). Nonetheless, both, mean values for sleep efficiency and onset in the first night of assessment, were within the overall recommendations for healthy sleep (Carskadon and Dement, 2011). Hence, it may be negligent to imply that sleep was heavily disturbed in the first night of assessment just because sleep parameters were more favorable in the second night's measurement. As six athletes showed higher sleep efficiency during the first night of assessment, overall suggestions may not be suitable for each individual athlete and it can be presumed, that some individuals are more affected by a first-night effect than others. Yet, in order to keep the measurement uncertainties to a minimum, a more conservative approach with at least two monitoring nights should be preferred, especially once before risky conclusions or hasty interventions are drawn.

Intention but likewise limitation of the study is, that athletes were not restricted in any kind, neither in their time spent in bed, nor in possible confounders (e.g., nutritional intake, training load, exposure to light) on the day before. This way, regular night's measurements with as little unfamiliar factors as possible could be secured. However, as no sleep researcher was present during the nights of assessment and data was interpreted subsequently, there is no evidence whether the participants actually tried to fall asleep at the start of measurement and whether they did sleep in the same sleeping environment during both nights. Besides, there is no proof that the second night actually represents a regular night of sleep and was not equally influenced by confounders. Potentially, participants may have felt the urge to spend more time in bed in night two, as they felt the need to make up for the sleep loss of the previous night. Thus, it needs to be stressed that the study setting should be interpreted in terms of a field study, rather than a controlled lab-based one. Still, as there was no other form of rewarding than an interpretation of the obtained sleep data, probability of compliance is believed to be very high.

To counteract these limitations, the use of different measurement devices in combination should be considered in future studies in order to grasp individual variances more efficiently. For example, portable PSG may be complimented by the use of sleep-logs or wrist-actigraphy for additional longitudinal monitoring (Hof zum Berge et al., 2020b). To further translate data from lab to practice, the implementation of a sleep diary or log on a regular basis may help to put measured data into perspective without objectively measuring every single night of sleep (Reed and Sacco, 2016; Kölling and Hof zum Berge, 2020). Accordingly, an athlete's sleep habits, architecture, and patterns must always be examined on an individual basis before implementing any interventions or structural changes (Driller et al., 2019; Hof zum Berge et al., 2021). Thus, different monitoring tools should not be considered as mutually exclusive. However, as portable PSG currently occurs to be the only reliable option to monitor sleep stage distribution outside the sleep-lab, it should be installed whenever the assessment of sleep architecture seems desirable (Hof zum Berge et al., 2020a).

Overall, it may be concluded that a first-night effect was found within this study sample and even though some individual athletes seem to be more resistant to the effect than others, general findings implicate that a second night of assessment should be recommended for future measurements if no other supporting information on the athletes says otherwise. Nonetheless, the documentation of confounding factors during future assessments may help to educate athletes on how to minimize these to optimize sleep hygiene and henceforth sleep quality (Driller et al., 2019; Vitale et al., 2019). At last, it should be noted that sleep is highly individual and even two nights of assessment should only be used as an indicator for further interventions and not as a clinical diagnosis without the assessment of a sleep medicine expert within a standardized set-up in the sleep-lab.

## Data Availability Statement

The raw data supporting the conclusions of this article will be made available by the authors, without undue reservation.

## Ethics Statement

The studies involving human participants were reviewed and approved by the Ethics Committee of the Faculty of Sport Science, Ruhr University Bochum. Written informed consent to participate in this study was provided by the participants' legal guardian/next of kin.

## Author Contributions

AHzB prepared the original manuscript, figures, and tables, conceived and analyzed the data, and interpreted the results. SJ and MK assisted with writing and editing the manuscript, figures, and tables. AHzB and SJ designed the experiment. All authors contributed to the article and approved the submitted version.

## Conflict of Interest

The authors declare that the research was conducted in the absence of any commercial or financial relationships that could be construed as a potential conflict of interest.
